# Provider Governance; A Basic Blackbox Seldom Looked at Properly: A Response to Recent Commentaries

**DOI:** 10.15171/ijhpm.2016.128

**Published:** 2016-09-24

**Authors:** Richard B. Saltman, Antonio Duran

**Affiliations:** ^1^Department of Health Policy and Management, Rollins School of Public Health, Emory University, Atlanta, GA, USA.; ^2^AllDMHealth, Seville, Spain.

The five commentaries on our initial article^1^ provide valuable insights into the complex process of re-framing the relationships involved in provider side decision-making in the health sector. These commentaries help focus more closely on the elements involved in the proposed shifting of institutional level decision-making; (*i*) away from full and exclusive dependence on governmental and political bodies, at either/or both national and regional level, one of the critical elements in the definition of governance; so that (*ii*) the new center of gravity is one or another structural arrangement simultaneously more targeted on patients and performance, as well as having greater potential to innovate effectively in its patient care routines and procedures.

One noticeable pattern in reading across the commentaries is in underscoring the importance of penetrating beyond formal macro and meso levels of governance, to insure that real change occurs in daily operational behavior at the micro clinical level, which of course is where patients receive care. This “shop-floor” impact was described as essential in order to achieve the desired degree of change in both hospital as well as primary care outcomes. The near unanimity of the comments is a particularly relevant coincidence with the original article.

Denis and Usher^2^ summarized this perception as *“good governance … also goes deep enough to influence clinical habits,”* further reinforcing this preferred organizational outcome in their last sentence which calls for “going deep and granular enough to foster good clinical habits.” Chinitz^3^ similarly concludes that good governance requires the “smooth meeting of top-down policy initiatives* … *with bottom-up innovations in micro-delivery systems involving front line staff.” Such *convergence* between clinical habits and good governance is of utmost relevance as it incorporates the notion of involving clinicians, usually seen as non-central elements in governance (see also below).

Back,^4^ writing about Swedish primary care, further reinforces this theme by noting that “it is* … *important to pay attention to the local organizational context, in terms of factors that can both prevent and facilitate a real change.” Also writing about the Swedish primary care reforms, Jeurissen and Maarse^5^ open a new angle of analysis (ownership of the facilities and the related organization) when they conclude that the major shift in visiting patterns created by new privately owned primary care providers could be contrasted with the fact that “earlier reforms to improve the primary care system *from within* had largely failed.”

Finally, although somewhat less emphatic about reinforcing shop-floor authority, Chanturidze and Obermann^6^ note that “good governance* … *should (permit) leeway for managers to be creative in countering unforeseeable needs and circumstances.”

A second point where all five commentaries agree is that additional research could help develop this area of study further, making the critical point that provider governance probably has not received sufficient attention as a research topic. One particular aspect that appeared to several commentators to require more research concerned the mechanisms and outcomes by which macro and meso level decision-making altered (or did not alter) micro level behavior, including but going beyond clinical behaviour.

Denis and Usher^2^ write that “The ‘black box” of clinical governance as a process between incentive and outcome needs to be unpacked to find the instruments that can support improvement at the micro level” implicitly in a complex though not unfolded relationship with the meso and macro levels above it. Chinitz^3^ similarly states in a straightforward manner that “more work needs to be done to assess the implications of governance shifts between the macro and meso levels on what happens at the micro level.”

Paralleling these concerns, the final sentence in the abstract by Back^4^ concludes that “this article calls for research* … *to capture everyday practice in-depth.” In a similar but closely related concern, Jeurissen and Maarse^5^ call for more information about the costs of running the new primary care model in Sweden, and about the seeming lack of interest across Europe in specifically not-for-profit models of innovative care delivery. That topic has major relevance for Europe, where innovative not-for-profit care has received comparatively less attention than other ownership and organizational modalities.

Where there was some difference between several of the commentaries and the original article was in the degree to which the formal official structures of government should continue to dominate over non-governmental private sector actors in the pursuit of governance outcomes. Several of the commentaries implicitly argue for continuation of a more dominant and coercive national and/or regional government decision-making function than did our initial article. Such continued reliance on formal government to resolve nearly all important dilemmas through restrictive legislation and/or regulation could be argued to undermine a fundamental distinction which underlines the analysis in our original article. The vision of formal government acting unilaterally as a command-and-control decision-maker is presented in our paper in explicit contrast to a broader, more inclusive, understanding of governance which combines the actions of both private and public decision-makers in the shaping of everyday provider behavior and activities through emerging relationships that are not captured by the word “government.”

For example, Chanturidze and Obermann^6^ state that “by ‘governance,’ we mean all ‘steering’ carried out by public bodies that seek to constrain, encourage, or otherwise influence acts of private and public parties,” thus, in practice eliminating all private sector actors (not-for-profit, group-practice, and small and large for-profit actors) from any effective governance role. Following up on this perspective, they subsequently call for “strong governance arrangements” by which formal public sector government controls continue unilaterally to determine all aspects of private sector health-related activity (p. 508).

Overall, there appears to be considerable overlap both of analysis and of suggestions for prospective next steps between the writers of these commentaries and our original article. It may be that working further within this overlapping area can help advance our ability to harness the concept of governance in the pursuit of more efficient and effective healthcare providers.

## Ethical issues

Not applicable.

## Competing interests

Authors declare that they have no competing interests.

## Authors’ contributions

Both authors contributed substantial written text to the article and reviewed the final version before publication.

## Authors’ affiliations

^1^Department of Health Policy and Management, Rollins School of Public Health, Emory University, Atlanta, GA, USA. ^2^AllDMHealth, Seville, Spain.
